# Cross-Over Pathogenic Bacteria Detected in Infected Tomatoes (*Solanum lycopersicum* L.) and Peppers (*Capsicum annuum* L.) in Bulgaria

**DOI:** 10.3390/pathogens11121507

**Published:** 2022-12-09

**Authors:** Yoana Kizheva, Georgi Georgiev, Deyan Donchev, Melani Dimitrova, Maria Pandova, Iliyana Rasheva, Petya Hristova

**Affiliations:** Department of General and Industrial Microbiology, Faculty of Biology, Sofia University, 1504 Sofia, Bulgaria

**Keywords:** cross-over pathogens, foodborne infections, *Solanum lycopersicum* L., *Capsicum annuum* L., antibiotic resistance

## Abstract

The ability of certain human pathogens to adapt to plants without losing their virulence toward people is a major concern today. Thus, the aim of the present work was the investigation of the presence of cross-over pathogenic bacteria in infected tomato and pepper plants. The objects of the study were 21 samples from seven different parts of the plants and three from tomato rhizosphere. In total, 26 strains were isolated, identified by MALDI-TOF, and phenotypically characterized. The PCR amplification of the *rpo*B gene was applied as an approach for the rapid detection of cross-over pathogens in plant samples. A great bacterial diversity was revealed from tomato samples as nine species were identified *(Leclercia adecarboxylata*, *Pseudesherichia vulneris*, *Enterobacter cancerogenus*, *Enterobacter cloacae*, *Enterobacter bugandensis*, *Acinetobacter calcoaceticus*, *Pantoea agglomerans*, *Pantoea ananatis*, and *Pectobacterium carotovorum*). Polymicrobial contaminations were observed in samples T2 (tomato flower) and T10 (tomato fruit). Five species were identified from pepper samples (*P. agglomerans*, *L. adecarboxylata*, *Pseudomonas* sp., *Pseudomonas putida*, and *Enterococcus* sp.). Antibiotic resistance patterns were assigned in accordance with EFSA recommendations. All isolates showed varying resistance to the tested antibiotics. The genetic basis for the phenotypic antibiotic resistance was not revealed. No genes for the virulence factors were found among the population. To our knowledge, this is the first overall investigation of tomato and pepper cross-over pathogenic bacterial populations in Bulgaria.

## 1. Introduction

Microorganisms found on the surface and inside plant tissues form a complicated network known as the plant’s microbial community, which interacts with the host plant and the surrounding environment [1]. Many members of these communities are beneficial for plant physiology and development, while others are plant and human pathogens [2]. Human pathogens can actively interact with plants and potentially colonize them as alternate hosts rather than merely contaminating plant surfaces [3]. It has been reported that some human pathogenic bacteria can cause rot and blight diseases in plants [4]. Likewise, some phytopathogenic bacteria also have the potential to infect human beings [5]. The ability of some pathogenic bacteria (phyto- or human) to cause physiological and morphological changes in novel hosts (from different kingdom) has led to the emergence of cross-kingdom pathogenicity. This pathogenesis can occur either directly or indirectly. Human pathogens may be indirectly transmitted to plants through the environment or with the help of any carrier, according to some theories. Phytopathogens can be transmitted to humans directly or indirectly through the environment [2,4].

Members of the family *Enterobacteriaceae* can be isolated from a variety of host species, representatives of all biological kingdoms, and can adapt to a wide range of environmental conditions. Recently, fresh products have been considered as the most prominent carrier of foodborne pathogens as over 35 serious disease outbreaks, linked with that kind of food, have been reported [6]. Most outbreaks have been connected to leafy greens, sprouting seeds, herbs, fruits of tomatoes, cucumbers, and peppers [7]. A significant outbreak of *Escherichia coli* STEC (Shiga toxin—producing *E. coli*) linked to bagged baby spinach occurred in the USA and Canada in 2006, altering our understanding of bacteria associated with plant foods [8]. This outbreak, which resulted in 206 documented instances of illness with three fatalities, was caused by wildlife contaminating spinach fields [9] and the pathogen’s capacity to spread in watercourses [10]. In November 2006, the CDC (Centers for Disease Control and Prevention) reported that a *Salmonella enterica* serovar Typhimurium outbreak caused 183 cases in 21 states. Analyses of the data collected by the investigators indicated that the consumption of infected tomatoes was the cause of this infection [11]. Cucumbers have also been linked to a multistate *Salmonella* epidemic in 2015, which has led to an increase in outbreaks [12]. The CDC reported an epidemic of listeriosis in December 2021 after the consumption of mixed salad greens [13]. It can be summarized that among the most common causative agents of foodborne outbreaks in edible crops are pathogens with zoonotic origin such as Shigatoxigenic *E. coli* (STEC), *S. enterica*, and saprophytes such as *Listeria monocytogenes*, which represent >80% of reported cases [14].

The understanding that some human pathogens are well-adapted to inhabiting plant niches requires a fundamental change in our perception of their ecological range and should be taken into account when developing control measures [3]. In fact, this may not come as a surprise considering that many other bacteria, which operate as endophytes, epiphytes, and pathogens, can successfully inhabit plants. Thus, the study of the plant microbiome should be extensively conducted in order for more knowledge of microbial plant communities to be obtained and summarized. Such overall investigations have been reported for tomato (*Solanum lycopersicum* L.) and pepper (*Capsicum annuum* L.) plants. A metagenomic research, revealing the epiphytic microbial community of healthy tomato plants, grown in a field with a previous *Salmonella* outbreak, was conducted [15]. Similarly, the composition of the plant and human pathogenic bacterial population of green pepper has also been reported in Saudi Arabia. In that investigation, *Proteus mirabilis* and *Klebsiella oxytoca* were identified from the pepper samples [16]. Furthermore, a strikingly significant percentage of the genomes of the plant pathogenic *Pectobacterium atrosepticum* and the plant-associated enterobacteria *Klebsiella pneumoniae* are shared with the genomes of human pathogenic enterobacteria, according to many studies of these organisms [17,18,19].

Cross-kingdom jumps have been observed most frequently in numerous *Enterobacteriaceae* and *Pseudomonadaceae* members. Despite being largely known as plant disease agents, some species in the genera *Pantoea*, *Burkholderia*, *Erwinia (Pectobacterium*), *Rhizobium*, and *Pseudomonas* can cause animal diseases. For instance, *K. pneumoniae* (endophyte) and *Pantoea agglomerans* (plant pathogen) have both been linked to opportunistic infections in mammals including humans [3]. *Dickeya dadantii* and *Pantoea ananatis* are plant pathogens that cause diseases in pea aphids and humans, respectively [20]. Similarly, *Salmonella*, *Enterobacter*, and *Shigella* species, which are typically considered as animal pathogens, can also infect plant hosts [21,22,23].

However, the examples provided do not represent the entire species diversity of cross-over pathogens in plants. We suppose that a significantly greater number of microorganisms can jump between biological kingdoms. Thus, plants with symptoms of bacterial diseases are an ideal natural source for the study of cross-over pathogen bacteria. Thus, the aim of the present work was to investigate the existence of cross-over pathogens inhabiting infected tomato and pepper plants and to study their antibiotic resistance patterns. To our knowledge, this is the first overall investigation of a tomato and pepper cross-over pathogenic bacterial population in Bulgaria.

## 2. Materials and Methods

### 2.1. Sample Collection and Isolation of Bacterial Strains

**Figure 1:**
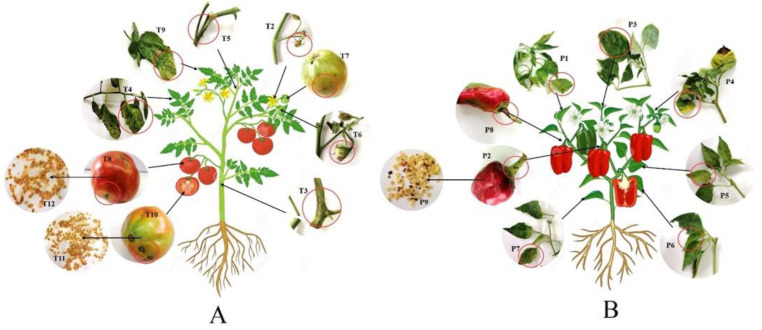
Plant samples. (**A**) Tomatoes samples: stems—T3, T5; leaves—T4, T9; flower—T2; fruits—T6, T7, T8, T10; and seeds—T11, T12; (**B**) Pepper samples: leaves—P1, P3, P4, P5, P6, P7; fruit stem–P2, P8; and seeds—P9. Samples T1, S1, S2, and S3 are not shown.

The location of sampling was a private crop field in the village of Piperkov Chiflik, Kyustendil Municipality, Kyustendil Province (GPS coordinates according Google Earth 42°16′33″ N 22°44′18″ E), southwestern Bulgaria. In the field, various crops (tomatoes, peppers, fruit trees, potatoes, etc.) irrigated with purified and chlorinated water were grown. All tomato and pepper plants grown in the field showed symptoms of microbial diseases. All 24 samples were randomly collected (21 from the aboveground parts of different tomato and pepper plants and three from tomato rhizospheres). Among them, 12 samples were collected from tomatoes and nine samples from peppers (Figure 1). The probes were collected in sterile plastic bags and transferred to the laboratory where they were immediately subjected to analysis.

All samples with disease symptoms were washed with sterile dH_2_O and handled with an alcoholic solution of iodine (2%) to eliminate the epiphytic bacterial population. The areas containing disease symptoms were cut off with a sterile scalpel and homogenized. Each sample was then immersed in saline and left for 30 min at room temperature with strong vortex every 5 min. The rhizosphere samples were prepared by mixing 10 g soil with 90 mL sterile saline. Seed samples (T11, T12, and P9) were separated from the fruits (T10, T8, and P2, respectively), homogenized, and mixed with sterile dH_2_O to obtain the work solutions. To isolate the cross-over pathogens, decimal dilutions of each sample were prepared and 100 μL of each dilution was spread on the surface of three selective media: Endo agar (Oxoid™ Endo Agar, CM0479B, Thermo Fisher Scientific, Waltham, MA, USA), Slanetz and Bartley (SB) agar (HiMedia, M612), and *Salmonella-Shigella* (SS) agar (HiMedia, M108). Three replications of plating were carried out. The colonies were distinguished based on their morphology. Single colonies obtained from each morphological type were isolated on Nutrient agar plates and cultured for 24 h at 37 °C to obtain pure cultures. Pure cultures were subsequently stored on non-selective media (Nutrient Agar, Merck, Darmstadt, Germany) at 4 °C for further analysis.

### 2.2. Phenotypic Characterization of the Bacterial Isolates

Phenotypic characterization of the obtained pure cultures was carried out according to the procedures described by Traub et al. [24]. The isolates were studied for: Gram’s reaction, Ct (catalase), and Ox (oxidase) production, M (motility), I (indole) production, MR (methyl red test), VP (Voges–Proskauer reaction), H_2_S, ornithine decarboxylase, L-lysine decarboxylase, citrate, urease, and gelatinase production, and the fermentation of glucose, arabinose, inositol, and lactose.

### 2.3. Extraction of DNA from the Isolated Bacterial Strains

The bacterial strains, isolated from different parts of the tomato and pepper plants, were cultivated in nutrient broth overnight at 37 °C. The resulted liquid cultures were used for genomic DNA isolation using an E.Z.N.A. Bacterial DNA Kit according to the supplier’s protocol (Omega, Bio-Tek, Inc., Winooski, VT, USA). The isolated DNA was purified by the DNA purification Kit (PCR/DNA Clean-up, GeneMatrix, EURx Ltd., Gdansk, Poland). The quantity of purified DNA was measured by BioDrop µLITE+ (100 ng/µL ± 20 ng/µL).

### 2.4. Detection of Enterobacterial Strains by PCR

All isolates were examined using the PCR technique to determine what proportion of the isolated endophytic bacteria belonged to the *Enterobacteriaceae* family. Partial *rpo*B genes were amplified with primer pairs *rpo*B/F (5’CAGGTCGTCACGGTAACAAG3’) and *rpo*B/R (5’GTGGTTCAGTTTCAGCATGTAC3’). The PCR procedure was performed as described by Fazzeli et al. [25]. All PCR reactions were performed with a PCR thermal cycler (Techne^®^ Prime, VWR International, Radnor, PE, USA) and the PCR products were visualized by 1.5% gel agarose electrophoresis. The following microorganisms were used as negative and positive controls to verify the discriminatory effect of the primers for *Enterobacteriaceae*: *Xanthomonas vesicatoria* NBIMCC 2427, *Xanthomonas gardneri* NBIMCC 8730, *Xanthomonas euvesicatoria* NBIMCC 8731, *Clavibacter michiganensis* subsp. *michiganensis* NBIMCC 2425, *Pseudomonas syringae* pv. *syringae* NBIMCC 2420, *Sphingomonas paucimobilis* NBIMCC 3733, *Pseudomonas syringae* pv. *tomato* NBIMCC 3374, *Pseudomonas putida* NBIMCC 1090, *Pseudomonas fluorescens* NBIMCC 8758, *Pseudomonas syringae* pv*. tomato* (isolate from infected tomato flower), *Pantoea agglomerans* (isolate from tomato rhizosphere soil), *Curtobacterium flaccumfaciens* (isolate from tomato stem), *Curtobacterium flaccumfaciens* (isolate from chili pepper leaf), *Rhizobium larrymoorei* (isolate from pepper fruit stem), *Rhizobium larrymoorei* (isolate from pepper leaf), *Escherichia coli* NBIMCC 8432, and *Salmonella enterica* NBIMCC 8691.

### 2.5. MALDI-TOF-MS

A matrix assisted laser desorption ionization-time of flight mass analysis (MALDI-TOF-MS) was performed for 26 pure cultures isolated from tomato and pepper plants. In brief, a single colony from an overnight culture was deposited on a polished steel MSP 96 target (Bruker Daltonics, Billerica, MA, USA) and overlaid with 1 μL of a saturated-cyano-4-hydroxycinnamic acid (HCCA) matrix solution (Bruker Daltonics). Unidentified strains were resubmitted using the extended protocol. Prior to the matrix, 1 μL of 70% formic acid was added to the bacterial spot and left dry out. Mass spectra were acquired using the microflex LT mass spectrometer (Bruker Daltonics) and analyzed with the research-use-only (RUO) software workflow and reference library MBT v. 4.1.100. All of the isolates were successfully identified with a score >1.7.

### 2.6. Search for Virulence Determinants

All isolates were screened for the presence of virulence determinants. The toxin gene (*hly*A), secretion gene (*inv*A), and invasion genes (*eae*A, *ipa*H) were amplified using primer pairs and a protocol described by Chen et al. [26].

### 2.7. Screening for Antibiotic Resistant Phenotypes

Antibiotic resistance patterns of endophyte microbiota, isolated from diseased tomato and pepper plants, were assigned to EFSA-recommended antibiotics for foodborne enterobacterial pathogens [27]. Antibiotic susceptibility of the isolates to ten antibiotics: ampicillin/sulbactam (10 µg/10 µg/disc), neomycin (10 µg/disc), chloramphenicol (30 µg/disc), streptomycin (30 µg/disc), kanamycin (30 µg/disc), tetracycline (30 µg/disc), gentamicin (200 µg/disc), trimethoprim (5 µg/disc), sulfamethoxazole (23.75 µg/disc), and nalidixic acid (30 µg/disc) was investigated using the disc-diffusion method in accordance with the Clinical and Laboratory Standards Institute (CLSI) guidelines on Nutrient agar [28]. After a 24-h anaerobic incubation at 37 °C, inhibition-zone diameters were determined and used as an indication to distinguish between susceptible and resistant isolates.

### 2.8. Screening for Genes Encoding Antibiotic Resistance

Total DNAs were used as templates to detect the resistance genes. The genes encoding antibiotic resistance to gentamicin (*aac*6′-*aph*2″), chloramphenicol (*cat*), tetracycline (*tet*M), β-lactam ase (*bla*Z), streptomycin (*aad*A, *aad*E), and macrolide (*mef*A) were examined by PCR with specific primers. Primers used for the amplification of the indicated genes and PCR reactions were performed according to the protocol of Guo et al. [29] and Liu et al. [30]. The PCR products were separated in 1.5% agarose gel electrophoresis.

## 3. Results

### 3.1. Isolation of the Bacterial Strains

A total of 21 samples from different parts of infected tomato and pepper plants with symptoms of microbial diseases and three from the tomato rhizosphere were randomly collected. Twenty-six bacterial strains were obtained from 13 plant samples after cultivation on the selective media used (Table 1). However, no potential enterobacterial or enterococcal bacteria were isolated from the rest of the 11 samples: T1, T4, T7, P1, P4, P5, P6, P9, S1, S2, and S3.

The presumptive endophyte bacterial population, isolated on the selective media used, was found to be more numerous in the tomato samples than in the pepper ones. The average number of bacteria isolated from tomato samples on Endo agar was 2.2 × 10^3^ CFU g^−1^ and 4.8 × 10^4^ CFU g^−1^ on SS agar. No growth of the SB medium was observed from the tomato samples. The bacterial population was detected in the flower (T2), stems (T3, T5), leaf (T9), fruits (T6, T8, T10), and seeds (T11, T12), which represent 75% of all of the tested tomato samples. The average number of bacteria isolated from the pepper samples on Endo agar was 2.5 × 10^2^ CFU g^−1^, 3.1 × 10^2^ CFU g^−1^ on SS agar, and 1.3 × 10^2^ CFU g^−1^ on the SB medium. Bacterial strains were isolated from the pepper fruit stems (P2, P8) and pepper leaves (P3, P7).

The colony morphology of the isolated bacterial strains (color, size, shape, and margins) was studied and 16 morphological types were differentiated. A single colony from each type was isolated to obtain pure cultures after three consecutive passages on the NB/NA medium. Some morphological variants were lost during purification, probably due to their inability to adapt to the laboratory environments or cultivation conditions. In the investigated bacterial population, colonies with homogeneous, granular, or mucoid structures were found. They had a convex, crater-shaped, or flat to teardrop-shaped profile, with a smooth or wavy (to rhizoid) margin, and a color varying in shades of cream, orange, and pink. Under the microscope, the majority (92.3%) of isolates were rod-shaped, and only 7.7% were spherical.

Cell sizes ranged from small to medium rods, with filaments observed in some cultures. According to the micro- and macromorphological characteristics, 18 strains were isolated from the tomato and eight strains from the pepper samples. The morphological diversity of strains from different plant parts varied significantly. The most morphological variants were obtained from the tomato flowers and fruits (five and four, respectively), whereas the tomato leaves had the least bacterial variability. In the pepper samples, five morphological variants were distinguished from the pepper fruit stem and two from the pepper leaves.

### 3.2. Phenotypic Characterization of Bacterial Isolates

A phenotypic characterization of 26 bacterial isolates was carried out. Approximately 92.3% (*n*  =  24) of the isolates were identified as Gram-negative and 7.7% (*n*  =  2) as Gram-positive. Next, 88.5% (*n*  =  23) were motility positive and 11.5% (*n*  =  3) were non-motile. In the studied population, 73.1% (*n*  = 19) did not have catalase activity, while 26.9% (*n*  = 7) were positive. Oxidase positive was 15.4% (*n*  = 4), while the remaining 84.6% (*n*  = 22) was negative. The ability to ferment glucose and arabinose was shown in 76.9% (*n*  = 20) and 69.2% (*n * = 18) of the isolates, respectively. Nine isolates (34.6%) were variable in the inositol utilization. Only 11.5% (*n * = 3) were fully fermented lactose, while the remaining 88.4% (*n*  = 23) were variable. Twenty isolates (76.9%) were urease-negative and 42.3% (*n * = 11) were lysine-decarboxylase-positive. All strains (*n * = 26) were capable of liquefying gelatin, and 73.1% (*n*  = 19) were citrate-positive (Table 2).

A crucial substrate for identifying Gram-negative bacteria is 2,6-butanediol, a substance that is produced during glucose fermentation from a precursor known as acetoin. The biochemical demonstration of acetoin is by the Voges–Proskauer (VP) reaction. A total of 53.8% (*n * = 14) of isolates were VP-positive and 46% (*n*  = 12) were VP-negative. On the medium containing iron salts, none of the isolates produced H_2_S, indicating that they were unable to convert sulfate-containing substances to sulfides. This result indicates that there were no *Salmonella* strains among the endophytic isolates. Tryptophanase is an enzyme found in isolates that can convert tryptophan to indole. Indole was detected only in four bacteria, and methyl red was detected in six isolates. Ornithine decarboxylase was present in 23 isolates, which enables them to convert ornithine to putrescine.

### 3.3. Detection of Enterobacterial Strains by PCR

**Figure 2:**
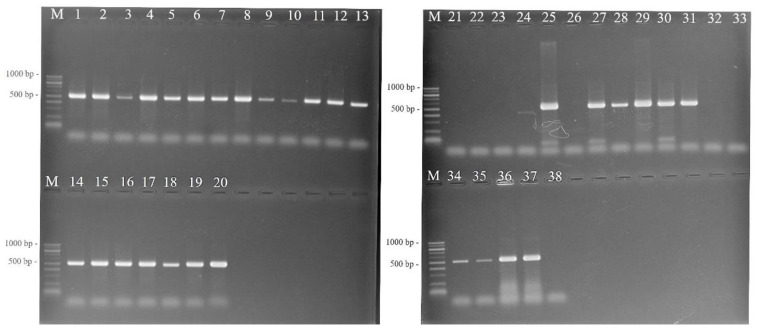
PCR amplification of the *rpo*B gene for rapid enterobacterial differentiation. Lanes—T 2.1; **2**—T 2.2; **3**—T 2.3; **4**—T 2.4; **5**—T 2.6; **6**—T 6.1; **7**—T 3.4; **8b**—T 5.4; **9**—T 9.2; **10**—T 8.4; **11**—T 10.1; **12**—T 10.2; **13**—T 10.3; **14**—T 11.2; **15**—T 12.3; **16**— P 3.5; **17**—P 2.4; **18**—P 8.5; **19**—P 8.6.; **20**–*E. coli* (tc*); **21**—*X. vesicatoria* (tc); **22**—*X. euvesicatoria* (tc); **23**—*X. gardneri* (tc); **24**—*C. michiganensis* subsp. *michiganensis* (tc); **25**—*P. syringae* pv*. syringae* (tc); **26**—*S. paucimobilis* (tc); **27**—*P. syringae* pv*. tomato* (tc)*;* **28**—*P. putida* (tc); **29**—*P. fluorescens* (tc); **30**—*P. syringae* pv*. tomato* (isolate); **31***—P. agglomerans* (isolate); **32**, **33**—*C. flaccumfaciens* (isolates); **34**, **35**—*R. larrymoorei* (isolates); **36**—*S. enterica* (tc); **37**—*E. coli* (tc); **38**—*Enterococcus* sp. *tc—type culture.

According to biochemical characterization, the majority of endophytic strains appear to be presumptive members of the *Enterobacteriaceae* family. The universal *rpo*B gene was amplified by PCR for family-level typing in order for rapid enterobacterial differentiation to be obtained. An amplification product with an expected length of 512 bp was generated by all Gram-negative isolates (*n* = 24) as opposed to the Gram-positive isolates (Figure 2). At this point of the study, all Gram-negative bacteria exhibited the *Enterobacteriaceae* PCR profile. However, the specificity of this primer set to identify members of the *Enterobacteriaceae* has not been widely validated in the plant microbial community [25]. Therefore, by using this combination of primers, we additionally amplified various phytopathogenic bacterial species (*X. vesicatoria*, *X. gardneri*, *X. euvesicatoria*, *C. michiganensis* subsp. *michiganensis*, *C. flaccumfaciens*, and *S. paucimobilis*) causing diseases on tomato and pepper plants. However, no specific amplification products were generated with the *rpo*B gene primers. Unexpectedly, species from different families including *R. larrymoorei*, *P. putida*, *P. fluorescens*, *P. syringae* pv. *syringae*, *P. syringae* pv. *tomato* produced an amplification product with an expected length of 512 bp.

### 3.4. Identification of Endophyte Isolates by MALDI-TOF-MS

The 26 endophytic bacterial strains were identified by the MALDI-TOF platform and the results are reported in Table 1. All of them belonged to 12 species from eight genera representing the following families: *Enterobacteriaceae*, *Erwiniaceae*, *Pectobacteriaceae*, *Moraxellaceae*, *Pseudomonadaceae*, and *Enterococcaceae*. The strains from *Enterobacteriales* order (73.1%) and *Enterobacteriaceae* family (42.3%) were prevalent. The species *Leclercia adecarboxylata* (23.1%, *n* = 6) and *P. agglomerans* (19.2%, *n* = 5) were dominant in the enterobacterial population. Strains of *L. adecarboxylata* were isolated from tomato flowers, tomato fruit, and pepper leaves. *P. agglomerans* representatives (*n* = 5) were discovered in the tomato plant fruit and seeds as well as pepper leaf. *P. ananatis* (*n* = 1) was isolated from tomato fruit. Five isolates of *Enterobacter* genus, belonging to the three species, *Enterobacter cloacae* (*n* = 2), *Enterobacter cancerogenus* (*n* = 2), and *Enterobacter bugandensis* (*n* = 1), were identified in the endophytic population. They were found in practically all tomato samples including flowers, fruits, leaves, and seeds, but not in pepper plants. The phytopathogenic bacterium *Pectobacterium carotovorum* was detected in sample T10.2 (tomato fruit). From the tomato stems, a strain belonging to the species *Pseudesherichia vulneris* was isolated. *Pseudomonas putida* and *Pseudomonas* sp., both members of the family *Pseudomonadaceae*, were found only in the pepper fruit stems. One strain belonging to the species *Acinetobacter calcoaceticus* (*Moraxellaceae* family) was isolated from the tomato flower (T 2.4.). The Gram-positive cocci (*n* = 2) were isolated from pepper fruit stems and identified as *Enterococcus* sp. Two isolates (T 2.5, and T 3.4) were not identified by the MALDI-TOF platform.

### 3.5. Searches for Virulence Determinants

We amplified certain genes involved in the virulence of some *Enterobacteriaceae* species such as *E. coli*, *S. enterica*, and *Shigella flexneri*, in order to establish their presence among our isolates. The toxin gene (*hly*A), secretion gene (*inv*A) and invasion genes (*eae*A, *ipa*H) were amplified. None of the isolates generated a positive amplification product.

### 3.6. Screening for Antibiotic Resistant Phenotypes and Genotypes

Antibiotic resistance patterns of the isolated bacteria were studied according to the EFSA-recommended antibiotics for enterobacterial foodborne pathogens. Out of the 11 species cross-over pathogens isolated in this study, 81.8% isolates were found to be resistant or moderately susceptible (intermediate) to gentamycin, 72.7% to chloramphenicol, 63.6% to neomycin and kanamycin, 54.5% to streptomycin and trimethoprim, 45.5% to sulfamethoxazole, and 36.4% to ampicillin/sulbactam. The rate of tetracycline resistance was low, as 90% of the isolates were susceptible. The isolates were susceptible or intermediate to nalidixic acid. The strain *E. bugandensis* was resistant or intermediate to the action of eight out of the ten tested antibiotics, while strain *A. calcoaceticus* was the most sensitive to the antibiotics applied (Table 3).

Since the majority of the strains were resistant to some antimicrobial substances, primer pairs for genes *aac* (6′)-*aph* (2″), *mef*A, *cat*, *aad*A, *aad*E, *bl*aZ, and *tet*M were used to amplify distinct resistant determinants. There were no positive amplifications for the genes evaluated in this investigation.

## 4. Discussion

The current investigation provides insight into the prevalence of the dominant members of endophytic bacterial communities in *Solanum lycopersicum* and *Capsicum annuum* capable of cross-jumping between hosts from different biological kingdoms. Cross-over pathogens have received a lot of attention recently as they have the potential to cause a significant number of foodborne illnesses, hospitalizations, and fatalities when contaminated fresh plant foods are consumed [31]. Vegetables have been considered as a major reservoir for opportunistic pathogens. In the case of endophytic existence in plant tissues, these bacteria cannot be removed by washing practices and thus they can pose a threat to human health. Moreover, evidence proving that cross-over pathogens have evolved to persist in plants as a normal part of their life cycle can be found in the literature [31].

In the present work, the identification of an endophytic population in diseased tomato and pepper plants revealed that 96% (*n* = 25) of isolated species were opportunistic pathogens (isolate T 10.2. is a typical phytopathogenic bacterium). The applied PCR approach for distinguishing the cross-over pathogens from phytopathogenic bacteria can be considered fairly reliable among the tested phytopathogenic species, causing diseases on tomatoes and peppers; only the representatives of the *Pseudomonadaceae* family generated the expected amplicon.

Shiga toxigenic *E. coli*, the different *S. enterica* serovars, and *L. monocytogenes* have been the most commonly described cross-pathogenic bacteria [5]. None of these microorganisms were found during our testing. In this study, two enterobacterial species, *L. adecarboxylata* and *P. vulneris*, which have not been previously described as cross-pathogens, were isolated from new hosts (infected tomato and pepper plants). Both species are opportunistic human pathogens phenotypically closely related to *E. coli*. Originally classified as *Escherichia adecarboxylata* and *Escherichia vulneris* within genus *Escherichia*, they were later divided into different genera of the *Enterobacteriaceae* family due to their genetic variability [32,33].

*L. adecarboxylata* is a widespread microbe found in aquatic habitats, soil, environmental sources, and animal specimens. It has been reported that some strains of the species, isolated from tomato rhizosphere, can have plant growth promoting activity [34]. To our knowledge, there are no data on the behavior of the bacterium in plant tissues during endophytic propagation. *L. adecarboxylata* can also be isolated from clinical specimens such as blood, urine, sputum, stool, and wound pus, and has been identified as an emerging opportunistic pathogen [35]. Endocarditis, urinary tract infections, pneumonia, catheter-related bacteremia, cellulitis, and bacterial peritonitis are examples where *L. adecarboxylata* is involved [36]. The bacterium was discovered in both healthy and immunocompromised individuals as part of polymicrobial infection, and less frequently of monomicrobial infection. The most frequent co-infecting organisms in polymicrobial infections with *L. adecarboxylata* clinical strains are *Enterococcus* species, *Cutibacterium acnes*, *Fusarium* species, *Staphylococcus epidermidis*, *K. oxytoca*, and *Pseudomonas aeruginosa* [36]. We found this dominant motile bacterium in the endophytic population in the flowers and fruits of tomatoes as well as in the leaves and stems of pepper. We also detected polymicrobial contamination in the studied plant samples. *L. adecarboxylata* coexisted with both *Enterococcus* and *Pseudomonas* in the pepper samples, successfully colonized tomato flowers along with several species of *Enterobacter*, and tomato fruits with the phytopathogens *P. agglomerans*, *P. ananatis*, and *P. carotovorum*. To our knowledge, this is the first report of *L. adecarboxylata* persisting in the aboveground parts of plants such as *S. lycopersicum* and *C. annuum*.

Most clinical isolates of *L. adecarboxylata* have been reported as susceptible to the most commonly applied antibiotics [37,38]. Beta-lactamase producing isolates and cephalosporin-resistant strains of *L. adecarboxylata* have been reported in only a few cases in human pathology [39]. In these rare cases, genetic determinants of resistance-encoding SHV-type beta-lactamases, *bla*, and *intl*1 genes were identified [40,41]. The unintentional consumption of infected food has recently been linked to an outbreak of carbapenem-resistant *L. adecarboxylata* [42].

These findings highlight the risk posed by foodborne cross-over pathogens. The first multidrug-resistant *Leclercia* species was isolated from an animal clinical case in India [43]. In our study, all *L. adecarboxylata* isolates showed resistance to streptomycin and gentamicin, moderate susceptibility to neomycin and kanamycin, and good susceptibility to ampicillin, tetracycline, chloramphenicol, sulfamethoxazole, and trimethoprim. The PCR did not identify resistance genes and virulence factors in wild strains of *L. adecarboxylata*. These results suggest a lower virulence of the wild-type *L. adecarboxylata* isolates compared to the clinical ones.

There have been few clinical reports of human infections with the opportunistic human pathogen *P. vulneris* [44,45,46]. The bacterium was first identified in humans in association with other bacteria including *Staphylococcus epidermidis*, *Staphylococcus aureus*, *Enterobacter* spp., *Acinetobacter lwoffii*, and *Cedecea neteri* [47]. It was later isolated from various clinical specimens such as feces, urine, sputum, vaginal swabs, and throat swabs, and was regarded as a colonizer [44]. In other research, *P. vulneris* was detected as a single pathogen in cases of osteomyelitis, urosepsis, intravenous catheter-mediated bacteremia, dialysis-related peritonitis, meningitis, and bacteremia in a patient with chronic lymphocytic leukemia [48,49,50]. In several of the clinical isolates, genes for LT enterotoxin and hemolysin (*hly*A) were found, showing the diarrheal potential of *P. vulneris* [51]. Microbial contamination of food and water are the main causes of diarrhea and invasive sepsis-inducing infection [51]. Our tomato stem isolate of *P. vulneris* T 5.4 did not contain hemolysin genes, indicating that it is incapable of causing diarrhea.

It has been reported that the clinical isolates of *P. vulneris* are susceptible to all classes of antibiotics [44,49,50]. There has only been one clinical report of *P. vulneris* developing an extended spectrum of β-lactamase, but resistance to chloramphenicol, ampicillin, tetracycline, and/or first- and second-generation cephalosporins has occasionally been described [52,53]. Compared to clinical isolates, our tomato isolate *P. vulneris* had a different antibiotic resistance profile. It was resistant to gentamicin and neomycin, intermediate to streptomycin, kanamycin, chloramphenicol, sulfamethoxazole, and nalidixic acid, and susceptible to ampicillin/sulbactam, trimethoprim, and tetracycline.

Representatives of three genera of phytopathogenic bacteria were also found in our samples: *Pectobacterium*, *Pantoea*, and *Enterobacter*. These phytopathogens cause diseases on trees, flowers, fruits, and vegetables that lead to quantitative and qualitative losses of crop production. For example, *P. carotovorum* is known as a common causative agent of soft rot and stem rot in tomatoes [54,55]. However, various members of the two genera, *Pantoea* and *Enterobacter*, are known opportunistic pathogens in humans, but infections usually require an immunocompromised host. The cross-over representatives of *P. agglomerans*, *P. ananatis*, *E. cancerogenus*, *E. cloacae*, and *E. bugandensis* species were found in the endophytic population of diseased tomato and pepper plants.

*P. ananatis* has rarely been identified as a human pathogen, but it has been reported to cause bacteremia [56]. The pathogenic *P. agglomerans* was isolated from cases of endophthalmitis, choledocholithiasis, bacteremia, pneumonia, osteomyelitis, urinary tract infection, and septicemia [57,58,59,60,61,62,63]. Additionally, connections between *P. agglomerans* and contaminated intravenous fluids, blood products, propofol, total parenteral nutrition, and powdered infant formula have been documented in other reports [58,64]. Pneumonia caused by *P. agglomerans* might be a life-threatening illness in young patients with underlying comorbidities. Clinical isolates of *P. agglomerans* have been reported to be resistant to carbapenems, ciprofloxacin, and piperacillin [65]. Our isolates of *P. agglomerans* were resistant to gentamicin and neomycin, intermediate to streptomycin, ampicillin/sulbactam, and kanamycin, and susceptible to the rest of the tested antibiotics. No genes determining antimicrobial resistance were found in their genomes.

*E. cancerogenus* has rarely been associated with human infections, and only a few cases of acute or chronic illnesses have been reported: osteomyelitis [66,67], urinary tract infection, infection of a traumatic cranial wound [68], bacteremia and pneumonia [69], open wound infection [70], hand traumatic cut infection, and infected hematoma after surgical treatment [71]. Our isolates showed a pattern of antibiotic susceptibility that was similar to patterns previously reported [67]. The T2.1 and T2.6 isolates of *E. cancerogenus* were resistant to neomycin and gentamicin, intermediate to streptomycin, kanamycin, sulfamethoxazole, trimethoprim, and chloramphenicol, and susceptible to ampicillin/sulbactam, tetracycline, and nalidixic acid. Some authors have reported that their clinical isolates were resistant or moderately susceptible to penicillin antibiotics due to the presence of β-lactamase genes [69]. Since the utilization of ampicillin/sulbactam inhibits the production of beta-lactamase, we were unable to confirm whether or not our isolates produced it.

*E. cloacae* is a naturally occurring component of the human intestinal flora that can be found in human and animal feces, water, plants, insects, and food. It is considered that this bacterium causes infection in immunocompromised patients. However, *E. cloacae* is a member of the *Enterobacter cloacae* complex (ECC), which also comprises other common nosocomial pathogens that can cause a wide range of diseases [72]. For example, *E. cloacae* has been reported as the main causative agent of various outbreaks in hospitals and it is known to cause respiratory and urinary tract infections. The bacterium is responsible for up to 5% of all hospital-acquired sepsis and nosocomial pneumonia cases, 4% of nosocomial urinary tract infections, and 10% of postoperative peritonitis cases [73]. Due to its opportunistic nature and capacity for antibiotic resistance, it has emerged as a significant pathogen and a global hazard [74]. The pathogenesis of *E. cloacae* is multifactorial and complex, since it involves a few virulence factors whose function is still unknown [75]. *E. cloacae’s* pathogenicity is attributed to the production of various virulence factors such as enterotoxins (cytotoxin similar to Shiga-like toxin II after the attachment to the epithelial cells), effector proteins secreted through the type III secretion system (T3SS), and the ability to destroy phagocytes [76,77,78]. Clinical isolates of *E. cloacae* have been reported to be resistant to ampicillin, cephalosporins, amoxicillin, and cefoxitin [79]. Surprisingly, the natural isolates of *E. cloacae* reported in this paper were susceptible to all tested antibiotics and only displayed resistance to chloramphenicol. We did not detect specific genes, coding the virulence determinants studied in our analyses, which may be due to their species specificity.

Recently, *E. bugandensis* has been identified as an enterobacterial species associated with severe clinical infection. The strain, obtained from a blood sample of an infected neonate, was resistant to amoxicillin/clavulanic acid, ampicillin, piperacillin/sulbactam, piperacillin-tazobactam, cefuroxime, cefalotin, cefoxitin, cefuroxime-axetil, cefpodoxime, cefotaxime, gentamicin, ceftazidime, ciprofloxacin, norfloxacin, tobramycin, tetracycline and trimethoprim/sulfamethoxazol [80]. In some studies, *E. bugandensis* has been classified among the most harmful *Enterobacter* species currently known [81]. The discovery that all antibiotic resistance genes are encoded on a common transmissible plasmid serves as a reminder of how easily pathogenic bacteria can develop a MDR (multidrug resistance) phenotype, which can have disastrous effects during outbreaks [81]. Unfortunately, our isolate of *E. bugandensis* can also be categorized as a MDR strain due to its resistance or intermediate sensitivity to eight out of 10 of the tested antibiotics. The strain showed susceptibility only to streptomycin and nalidixic acid. However, this resistance is intrinsic as no genes responsible for it have been discovered.

*Acinetobacter* species are typically found in humid places including moist soil, lakes, water treatment facilities, fish farms, sewage, and even seawater [82]. *Acinetobacter* is represented by more than 50 species, but most of them are nonpathogenic, environmental organisms. *Acinetobacter baumannii* is the most frequent infection agent, followed by *A. calcoaceticus* and *Acinetobacter lwoffii* [83]. Some clinically relevant species such as *A. calcoaceticus* are a widespread component of the typical human microflora and have been found on vegetables, meat, and dairy products [84,85]. *A. calcoaceticus* has been considered as a common pathogen associated with a wide range of diseases including pneumonia, bacteremia, urinary tract infections, and burn wound infections [86]. Numerous clinical settings have documented *A. calcoaceticus* nosocomial infection outbreaks [87]. However, antimicrobial resistance is the main factor affecting clinical outcomes. Antibiotic resistance genes such as carbapenemases and extended-spectrum β-lactamases (ESBLs) are frequently reported among the environmental isolates of the species [82]. Thus, they may serve as crucial environmental reservoirs for the emergence of clinically relevant strains. The strain of *A. calcoaceticus*, characterized in our investigation, was isolated from tomato flower, which could be a prerequisite for its persistence in the mature fruit. The isolate was susceptible to most antibiotics tested, but resistant to trimethoprim and chloramphenicol. No genes responsible for this resistance were found in the genome of *A. calcoaceticus*. There are a few different reasons for the established phenotypic resistance. The few and relatively small porins in *Acinetobacter*’s outer membrane contribute to its broad resistance to antibiotics. *Acinetobacter* has a lower permeability to antibiotics compared to other Gram-negative bacteria due to the reduced outer membrane porin concentration [88]. Additionally, *Acinetobacter* has one or more active efflux systems (such as AdeABC and AdeIJK) expressed constitutively at low levels [89]. There are few therapeutic alternatives as a result of the interaction between constitutive efflux and low permeability to antimicrobials, which leads to intrinsic resistance to a wide range of antibiotics [90].

*P. putida* is a member of the *Pseudomonas* species fluorescent group, widely discovered in the environment and previously regarded to possess weak virulence [91]. *P. putida* can colonize humid, inanimate hospital surfaces, leading to nosocomial infections, particularly in patients with impaired immune systems and those who use catheters or other medical devices [92]. There are also reports of bloodstream infection outbreaks linked to contaminated fluids [93,94,95]. The *P. putida* strain isolated in this study from pepper fruit stem showed resistance to different classes of antibiotics (five out of 10 tested) and ranked second in antibiotic resistance after *E. bugandensis*.

## 5. Conclusions

Most strains isolated in the present study had the profile of opportunistic pathogens for humans. Some of them can participate in polymicrobial infections and show diverse phenotypic antibiotic resistance to major classes of antibiotics. Our results showed that some species were found in tomato seeds, flowers, and fruits (*E. cloacae*, *P. agglomerans*, and *L. adecarboxylata*), which could indicate their distribution pathway in plants. Thus, we can speculate that seeds can be considered as one possible source of this contamination.

In conclusion, the ability of certain cross-over pathogens to adapt to plants without losing their virulence toward people is a major concern today. Detailed reports on the endophytic bacterial community of vegetables and fruit, survival rate of pathogens in non-host environments, etc., must be determined to adequately implement actions for control of these outbreaks.

## Figures and Tables

**Table 1:** Diversity of the endophytic bacterial isolates from *Solanum lycopersicum* and *Capsicum annuum*.

Bacterial Isolates	Source Plant Parts	Isolation Media	Cell Shape	Gram Stain	Taxonomic Identification of Bacterial Isolates by MALDI-TOF	ID Score Value
T 2.1.	Tomato flower	Endo agar	Short rods	negative	*Enterobacter cancerogenus*	1.97.
T 2.2.	Tomato flower	Endo agar	Short rods	negative	*Enterobacter cloacae*	1.92
T 2.3.	Tomato flower	Endo agar	Short rods	negative	*Leclercia adecarboxylata*	2.29
T 2.4.	Tomato flower	Endo agar	Short rods	negative	*Acinetobacter calcoaceticus*	1.81
T 2.5.	Tomato flower	Endo agar	Short rods	negative	*unidentified isolate*	1.23
T 2.6.	Tomato flower	SS agar	Short rods	negative	*Enterobacter cancerogenus*	1.95
T 6.1.	Tomato flower	Endo agar	Short rods	negative	*Leclercia adecarboxylata*	1.93
T 3.4.	Tomato stem	Endo agar	Short rods	negative	*unidentified isolate*	1.34
T 5.4.	Tomato stem	Endo agar	Short rods	negative	*Pseudesherichia vulneris*	1.92
T 9.2.	Tomato leaf	Endo agar	Short rods	negative	*Enterobacter bugandensis*	1.84
T 8.4.	Tomato fruit	Endo agar	Short rods	negative	*Pantoea agglomerans*	2.05
T 10.1.	Tomato fruit	Endo agar	Short rods	negative	*Pantoea ananatis*	1.97
T 10.2.	Tomato fruit	Endo agar	Short rods	negative	*Pectobacterium carotovorum*	2.06
T 10.3.	Tomato fruit	Endo agar	Short rods	negative	*Pantoea agglomerans*	2.02
T 10.4.	Tomato fruit	Endo agar	Short rods	negative	*Leclercia adecarboxylata*	2.43
T 11.2.	Tomato seed	Endo agar	Short rods	negative	*Pantoea agglomerans*	1.99
T 12.3.	Tomato seed	Endo agar	Short rods	negative	*Pantoea agglomerans*	1.89
T 12.4.	Tomato seed	Endo agar	Short rods	negative	*Enterobacter cloacae*	2.18
P 3.5.	Pepper leaf	Endo agar	Short rods	negative	*Pantoea agglomerans*	2.11
P 7.3.	Pepper leaf	Endo agar	Short rods	negative	*Leclercia adecarboxylata*	2.39
P 7.4.	Pepper leaf	Endo agar	Short rods	negative	*Leclercia adecarboxylata*	1.91
P 2.4.	Pepper fruit stem	SS agar	Short rods	negative	*Pseudomonas* sp.	2.19
P 8.5.	Pepper fruit stem	SS agar	Short rods	negative	*Pseudomonas putida*	1.89
P 8.6.	Pepper fruit stem	Endo agar	Short rods	negative	*Leclercia adecarboxylata*	2.08
P 8.7.	Pepper fruit stem	SB agar	cocci	positive	*Enterococcus* sp.	1.98
P 8.8.	Pepper fruit stem	SB agar	cocci	positive	*Enterococcus* sp.	2.01

**Table 2:** Biochemical characterization of the endophytic bacterial isolates.

Bacterial Isolates	Glucose	Arabinose	Inositol	Lactose	L-lysine Decarboxylase	Urease	Gelatin	Citrate	VP	H_2_S	Methyl-Red	Ornithine	Indole
T 2.1.	+	+	−	V	+	V	+	+	+	−	−	+	−
T 2.2	+	+	−	V	+	−	+	+	+	−	−	+	−
T 2.3	+	+	−	V	−	−	+	−	−	−	+	+	+
T 2.4	+	−	−	V	V	V	+	+	+	−	−	+	−
T 2.5.	+	+	V	V	−	−	+	−	−	−	+	+	−
T 2.6	+	+	−	V	V	−	+	+	+	−	−	+	−
T 6.1	−	−	−	V	+	−	+	+	−	−	−	+	−
T 3.4.	+	+	−	V	+	−	+	+	+	−	−	+	−
T 5.4	+	+	−	+	+	−	+	−	−	−	+	+	−
T 9.2	+	+	−	V	−	−	+	+	+	−	−	+	−
T 8.4.	+	+	V	V	−	−	+	+	+	−	−	+	−
T 10.1	+	+	V	+	V	−	+	+	+	−	−	+	+
T 10.2	−	+	V	+	−	−	+	+	−	−	−	+	−
T 10.3	+	+	−	V	−	−	+	V	+	−	−	+	−
T 10.4	−	−	−	V	+	−	+	+	−	−	−	+	−
T 11.2	+	+	V	V	−	−	+	+	+	−	−	+	−
T 12.3	+	+	V	V	−	−	+	V	+	−	+	+	−
T 12.4	+	+	−	V	+	+	+	+	+	−	−	+	−
P 3.5	+	−	V	V	−	−	+	−	+	−	−	+	−
P 7.3.	+	+	V	V	+	V	+	+	+	−	−	+	+
P 7.4	−	−	−	V	+	−	+	+	−	−	−	+	−
P 2.4.	−	−	−	V	+	+	+	+	−	−	−	−	−
P 8.5.	−	−	−	V	+	−	+	+	−	−	−	+	−
P 8.6	+	+	V	V	−	−	+	−	−	−	+	+	+
P 8.7.	+	+	−	V	−	−	+	+	−	−	−	−	−
P 8.8	+	−	−	V	−	V	+	+	−	−	+	−	−

**Table 3:** Antibiotic resistance patterns of cross-over pathogens isolated from infected tomato and pepper plants.

Bacterial Strains	Streptomycin	Tetracycline	Ampicillin/Sulbactam	Neomycin	Kanamycin	Sulfamethoxazole	Chloramphenicol	Trimethoprim	Nalidixic Acid	Gentamycin
*Leclercia* *adecarboxylata*	R	S	S	I	I	S	S	S	S	R
*Enterobacter* *cancerogenus*	I	S	S	R	I	I	I	I	S	R
*Enterobacter* *cloacae*	S	S	S	S	I	S	R	S	S	S
*Enterobacter* *bugandensis*	S	R	R	I	I	R	I	R	S	R
*Pantoea* *agglomerans*	I	S	I	R	I	S	S	S	S	R
*Pantoea* *ananatis*	I	S	S	R	S	S	S	S	S	R
*Pseudoesherichia vulneris*	I	S	S	R	I	I	I	S	I	R
*Pectobacterium carotovorum*	R	S	S	R	I	S	I	R	I	R
*Pseudomonas putida*	S	S	R	S	S	R	R	R	I	R
*Pseudomonas* spp.	S	S	R	S	S	R	R	R	I	R
*Acinetobacter* *calcoaceticus*	S	S	S	S	S	S	R	R	S	S

R—resistant; S—susceptible; I—intermediate.

## Data Availability

Not applicable.
